# Improving the Prediction of Local Drug Distribution Profiles in the Brain with a New 2D Mathematical Model

**DOI:** 10.1007/s11538-018-0469-4

**Published:** 2018-08-08

**Authors:** E. Vendel, V. Rottschäfer, E. C. M. de Lange

**Affiliations:** 1 Mathematical Institute, Leiden University, PO Box 9512, 2300 RA Leiden, The Netherlands; 2 Division of Systems Biomedicine & Pharmacology, Leiden Academic Centre for Drug Research, PO Box 9502, 2300 RA Leiden, The Netherlands

**Keywords:** Brain extracellular fluid, Pharmacokinetics, Mathematical model, Drug binding, Drug transport

## Abstract

The development of drugs that target the brain is very challenging. A quantitative understanding is needed of the complex processes that govern the concentration–time profile of a drug (pharmacokinetics) within the brain. So far, there are no studies on predicting the drug concentration within the brain that focus not only on the transport of drugs to the brain through the blood–brain barrier (BBB), but also on drug transport and binding within the brain. Here, we develop a new model for a 2D square brain tissue unit, consisting of brain extracellular fluid (ECF) that is surrounded by the brain capillaries. We describe the change in free drug concentration within the brain ECF, by a partial differential equation (PDE). To include drug binding, we couple this PDE to two ordinary differential equations that describe the concentration–time profile of drug bound to specific as well as non-specific binding sites that we assume to be evenly distributed over the brain ECF. The model boundary conditions reflect how free drug enters and leaves the brain ECF by passing the BBB, located at the level of the brain capillaries. We study the influence of parameter values for BBB permeability, brain ECF bulk flow, drug diffusion through the brain ECF and drug binding kinetics, on the concentration–time profiles of free and bound drug.

## Introduction

The development of drugs that target the brain and reach the target site in adequate levels is very challenging. Therefore, a quantitative understanding is needed of the highly complex processes that govern the concentration–time profile of a drug (pharmacokinetics) within the brain, and particularly at the brain target site. These include the transport of a drug between the blood and the brain and the distribution of a drug within the brain.

The transport of a drug from the blood into the brain is tightly regulated by the blood–brain barrier (BBB). As the main barrier of the brain, the BBB separates the blood from the brain extracellular fluid (ECF), which may cause the drug concentration–time profiles in blood and brain to be substantially different from each other (Hladky and Barrand 2014).

Although the BBB is a major determinant of the drug concentration within the brain, the fate of a drug within the brain cannot be explained solely by BBB transport. Also the factors that govern the distribution of the drug within the brain need to be considered. After crossing the BBB, the drug resides in the brain ECF. The brain ECF is the fluid surrounding the neural cells and is important in the supply of nutrients, waste removal and intercellular communication, see, e.g. Lei et al. (2017) for a recent review on this topic. In the brain ECF, drug transport occurs by diffusion and brain ECF bulk flow. Relatively to free diffusion through water, diffusion of a drug through the brain ECF is less effective, because of the space occupied by brain cells as well as the extracellular matrix. This is what is called tortuosity (Nicholson et al. 2011; Hladky and Barrand 2014). Tortuosity differs between drugs, because of their different size and deformability and the drug-specific interaction with the extracellular matrix (Nicholson et al. 2011).

The brain ECF bulk flow is another means of drug transport within the brain (de Lange and Danhof 2002; Cserr and Ostrach 1974). This movement of the brain ECF and its constituents is the result of a pressure gradient across the brain ECF (Abbott 2004; Han et al. 2012; Hladky and Barrand 2014). Changes in the brain ECF bulk flow may play a role in brain diseases and may affect drug distribution (Marchi et al. 2009, 2016).

While being transported by diffusion and by brain ECF bulk flow, drugs within the brain may associate with binding sites. Here, free drug associates with a free binding site with a certain on-rate, while the drug-binding site complex dissociates with a certain off-rate. Understanding these drug binding kinetics is very relevant, as the binding of a drug to its target determines its effect. The impact of this drug–target binding could be affected by drug binding to non-specific binding sites, which reduces the concentration of free drug that is available to bind to its target. Specific binding sites are mostly located on the brain cell surface or within the brain cells, but may also be located in the brain ECF, like enzymes. There are typically more non-specific binding sites than specific binding sites present, while the binding of a drug to non-specific binding sites is generally weaker than its binding to specific binding sites.

The brain is far from a homogeneous tissue, and many factors may result in local differences in drug concentration. For example, the density of binding sites within the brain can differ substantially between different regions. Recently, it has been shown that differences in target density in combination with target association and dissociation kinetics may influence local drug distribution (de Witte et al. 2016). Such changes in local pharmacokinetics are therefore important to consider. Altogether, a deeper insight is needed on how both drug-specific parameters (e.g. BBB permeability) and system-specific parameters (e.g. brain ECF bulk flow) influence the local concentration–time profiles of drugs within the brain. There are several studies that have focused on one or more of aspects of the distribution of a drug within the brain, which we discuss in “Literature”. However, none of these models contains all processes that govern spatial variability in drug concentration. Thus, there is a need for an integrative approach of these processes in order to ultimately predict local drug concentration–time profiles in the brain, as the drive of the effect of the drug.

As a next step towards such understanding, we formulate a 2D brain tissue unit model, where drug transport across the BBB and within the brain ECF, and the interaction of a drug with both specific (target) and non-specific binding sites are incorporated. This combination of properties of the model makes it the first in its kind.

**Literature**

A model that fully describes the distribution of a drug within the brain does not yet exist. In this section, we highlight some earlier models on the distribution of compounds within the brain. Here, a compound may be an exogeneous compound, such as a drug, or an endogeneous compound, such as a metabolite. The existing models generally focus on just one or two of the following properties (Table 1):

1. The exchange of a compound between several compartments related to the brain.
2. The transport of a compound within the brain ECF by diffusion and brain ECF bulk flow.
3. The binding kinetics of a compound. Binding kinetics describe the concentration–time profiles of not only free, but also bound compound, as determined by the rates of binding and unbinding of free compound to a binding site. Here, a distinction is made between specific binding, in which a compound binds to a specific target site, and non-specific binding, in which a compound binds to a non-specific, off-target binding site.
  In Table 1, we highlight several examples of models that include one or two of these processes. The exchange of a compound between several compartments can be described by compartmental models (Stevens et al. 2011; Westerhout et al. 2012, 2013, 2014; Ball et al. 2014; Gaohua et al. 2016; Yamamoto et al. 2017a, b). The compartments described by these models can represent the blood, a tissue (e.g. the brain) or the components of a tissue (e.g. the brain ECF). Moreover, they can represent different states of a compound, such as a bound and an unbound state. Within each compartment, the concentration–time profile of a compound is described by ordinary differential equations (ODEs).

**Table 1:** Existing models on compound distribution within the brain

Model properties	BBB transport	Compartmental exchange	Transport within the (brain) ECF		Binding kinetics	
References			Diffusion	Bulk flow	Specific	Non-specific
Compartmental exchange						
Westerhout et al. (2012, 2013, 2014), Stevens et al. (2011) and Yamamoto et al. (2017a, b)	*	${+}$	${-}$	${-}$	${-}$	${-}$
Kielbasa et al. (2009)	*	${+}$	${-}$	${-}$	**	**
Ball et al. (2014) and Gaohua et al. (2016)	${+}$	${+}$	${-}$	${-}$	**	**
Transport within the (brain) ECF						
Benveniste et al. (1989), Chen et al. (2002), Hrabětová et al. (2003), Hrabe et al. (2004) and Xiao et al. (2015)	${-}$	${-}$	${+}$	${-}$	${-}$	${-}$
Linninger et al. (2005), Linninger et al. (2008), Raghavan and Brady (2011) and García and Smith (2009)	${-}$	${-}$	${+}$		**	**
Dykstra et al. (1992) and de Lange et al. (1995)	*	${-}$	${+}$	${-}$	${-}$	${-}$
Nicholson (1995, 2001) and Morrison et al. (1994)	*	${-}$	${+}$	${+}$	**	**
Levin et al. (1980) and Robinson and Rapoport (1990)	$+$	${-}$	${+}$	${-}$	${-}$	${-}$
Saltzman and Radomsky (1991) and Patlak and Fenstermacher (1975)	${+}$	${-}$	$+$	${-}$	**	**
Binding kinetics						
Pan et al. (2013) for review	${-}$	${-}$	${-}$	${-}$	${+}$	${-}$
de Witte et al. (2016)	${-}$	${+}$	${-}$	${-}$	${+}$	${-}$
Combination of properties						
Collins and Dedrick (1983) and Calvetti et al. (2015)	*	${+}$	${+}$	${-}$	${-}$	${-}$
Bassingthwaighte et al. (1989)	${-}$	${+}$	${+}$	${-}$	${-}$	${-}$
Trapa et al. (2016)	${+}$	${+}$	${-}$	${+}$	${-}$	**
Tan et al. (2003) and Ehlers and Wagner (2015)	${-}$	${+}$	${+}$	${+}$	${-}$	${-}$
Jin et al. (2016)	${-}$	${+}$	${+}$	${+}$	${-}$	${-}$
Zhan et al. (2008)	${-}$	${+}$	${+}$	${+}$	**	**
Tzafriri et al. (2012) and McGinty and Pontrelli (2015, 2016)	${-}$	${+}$	${+}$	${+}$	${+}$	${+}$

The existing models on compound distribution within the brain have different properties. They may focus on compartmental exchange, transport in the (brain) ECF or binding kinetics. Some models focus on a combination of these processes. When a process is included in a model, this is, unless otherwise specified, indicated by a $+$ and when it is not, it is indicated by a −

*BBB transport is modelled as a capillary exchange rate between the blood and the brain ECF (Yamamoto et al. 2017a; de Lange et al. 1995; Calvetti et al. (2015)) or as a term that describes the loss of free compound from the system to the blood plasma (Nicholson 2001)

**Binding is taken into account by a term that describes the loss of free compound from the system to binding sites or by a term on binding affinity. However, binding *kinetics*, involving the concentration–time profiles of both free and bound compound and the association and dissociation rates of binding, are not considered

Recently, a compartmental model of the brain has been developed to provide understanding on the time-dependent drug distribution into and within the brain (Yamamoto et al. 2017a). There, the concentration–time profiles of nine drugs with highly distinct physico-chemical properties are described for multiple physiological compartments of the central nervous system (CNS). These compartments include the blood, the brain ECF, the brain intracellular fluid (ICF) and the cerebrospinal fluid (CSF). The CSF is connected to both the blood and the brain ECF and plays an important role in brain homoeostasis. The CSF is widely distributed (it is located in the ventricles of the brain, the subarachnoid space, which covers the brain and the spinal cord) and therefore is described in the model by four different compartments. In addition, two peripheral compartments are added to the model to include drug exchange with non-brain compartments. The model allows for an adequate prediction of the concentration–time profile of the drugs in the several compartments. However, in this and in other typical compartmental models, the brain ECF is considered homogeneous, while spatial concentration differences may exist. These concentration differences may arise due to various factors, including local differences in drug–target concentration and local disease. Therefore, to get more insight into the spatial distribution of a drug within the brain, models with other properties are necessary.

The transport of compounds through the brain ECF has extensively been described by the group of Nicholson (e.g. Syková and Nicholson 2008; Nicholson 2001). They have proposed a diffusion equation to model the transport of drugs through the brain ECF for drugs administered directly into the brain (see Nicholson 2001 for a thorough review on this topic). The diffusion equation includes terms for drug transport by diffusion and brain ECF bulk flow as well as terms that describe the drug entry into and drug loss from the brain ECF by BBB transport, metabolism and drug binding. However, the model lacks a more detailed description of these processes, such as: a more explicit description of BBB transport that includes the BBB permeability and the drug concentrations in the blood plasma and the brain ECF and a more explicit description of drug binding that includes drug binding kinetics and a distinction between binding to specific and non-specific binding sites.

The diffusion equation is used in many studies on drug distribution within the brain ECF (Nicholson 1995; de Lange et al. 1995; Chen et al. 2002; Saltzman and Radomsky 1991). It can be used to predict the local distribution of a drug after its application (Saltzman and Radomsky 1991; Morrison et al. 1994; Patlak and Fenstermacher 1975; Dykstra et al. 1992). For example, de Lange et al. (1995) use a radial diffusion equation to describe the spatial distribution of a drug after the local perfusion of drug via a cylindrical microdialysis probe. They fit the model to radial distribution data that have been determined for two drugs with different BBB transport properties but similar effective diffusion coefficients. Successful fits indicate the importance of BBB transport as well as diffusion through the brain ECF.

The mentioned models lack descriptions of drug binding kinetics. These are crucial to understand, as the binding of a drug to its target is what makes the drug exert its effect. Drug binding is commonly measured by the drug affinity, which is a measure of the strength of the interaction between the drug and its target. Since the introduction of the drug residence time that measures the time a drug interacts with its target and the appreciation of the fact that a drug can only elicit its effect during the period that it is bound to its target (Copeland et al. 2006; Swinney 2004), the kinetics of drug binding have gained more interest. As reviewed in Pan et al. (2013), the association and dissociation rates of drug binding as well as the concentrations of free drug and its binding sites determine the concentration–time profiles of free and bound drug. Earlier studies on drug binding kinetics have focused mostly on the drug dissociation rate as a determinant of the time course and duration of drug–target interactions, but a recent study has shown that the rate of association of a drug to its target can be equally important to determine the time course and duration of drug–target interactions (de Witte et al. 2016). There, drug binding kinetics are integrated in a compartmental model, existing of two compartments representing the bound and unbound state of the drug. In addition, in a second model an additional compartment is introduced to include drug distribution into and out of the tissue.

More studies exist that integrate more of the discussed properties into one model. For example, the distribution of a compound within the brain can be described by both compartmental exchange and transport through the brain ECF (Tan et al. 2003; Zhan et al. 2008; Calvetti et al. 2015; Ehlers and Wagner 2015; Jin et al. 2016). In Calvetti et al. (2015), a 3D model of brain cellular metabolism with spatial resolution of the location of the synapse relative to the brain capillaries demonstrates the importance of spatial distribution. There, it is found that the time course of metabolic fluxes and concentrations of compounds related to metabolism in brain cells is affected significantly by the distance between the cells and the brain capillaries. Another study that emphasises the importance of spatial distribution, although not concerning the brain, is the model by Bassingthwaighte et al. (1989). This model includes the exchange between the blood plasma, endothelial cells, parenchymal cells and the (non-brain) ECF as well as the transport within these compartments. It is shown that changes in parameters related to local blood flow, metabolism and binding influence the exchange of solute between the compartments. Moreover, it is demonstrated that the distance to the capillary influences the local concentration profile of solute in the tissue.

Models on drug distribution within the brain are particularly relevant when they are coupled to drug binding to its target, because only then, more knowledge about the effect of the drug can be acquired. To our knowledge, no studies exist where drug distribution within the brain ECF and drug binding kinetics are integrated in one model. In a recent work by McGinty and Pontrelli (2016) that focuses on local drug delivery to biological tissue such as the arterial wall, the diffusion equation that describes the concentration changes in free drug in the (non-brain) ECF is coupled to two ODEs that describe the concentration changes in drug bound to specific and non-specific binding sites (Tzafriri et al. 2012; McGinty and Pontrelli 2015, 2016). This work is one of the few studies that make a distinction between drug binding to specific binding sites and drug binding to non-specific binding sites. However, as this work does not focus on the brain, it lacks a description of transport across a tight barrier, such as the BBB. A work that combines the transport of a drug within the (brain) ECF and drug binding kinetics into one model (like in McGinty and Pontrelli 2015, 2016), but also explicitly describes how a drug enters the brain by crossing the BBB, is still lacking.

**Our approach**

None of the currently existing mathematical models on drug distribution within the brain includes all of the discussed properties, including compartmental exchange, drug transport through the brain ECF and drug binding.

Here, we introduce a 2D model in which the essentials of all of these processes are integrated. With the aim of ultimately developing a comprehensive 3D model based on 3D building blocks or units, we started to develop a single-unit 2D model that provides understanding of the distribution of a drug within the brain. This 2D model allows the investigation of the effect of several parameters, related to blood–brain exchange (BBB transport), transport within the brain ECF and binding, on the distribution of a drug within the brain.

We focus on the local drug concentration within the brain, based on a physiological representation of the brain, in which a (2D) piece of brain tissue is surrounded by the brain capillaries, where the BBB is located. Here, drug exchanges between the blood plasma and the brain ECF. Within this piece of brain tissue, drug is distributed through the brain ECF by diffusion in the presence of the brain ECF bulk flow. Moreover, drug distributes by binding to both specific and non-specific binding sites. This piece of brain tissue could be considered the smallest building block of the brain in terms of drug distribution, and therefore, we call it the brain tissue unit.

We use a partial differential equation (PDE) that accounts for diffusion through the brain ECF combined with brain ECF bulk flow to describe the change in free drug concentration in the brain ECF. To include drug binding to specific binding sites, we couple this PDE for free drug concentration to an ODE that describes the change in concentration of drug bound to specific binding sites. To incorporate non-specific binding in the model, we also couple this PDE to an ODE that describes the change in concentration of drug bound to non-specific binding sites. With our boundary conditions, we explicitly model drug transport across the BBB. They reflect how a drug enters and leaves the brain ECF across the BBB by describing the BBB permeability, i.e. the rate of drug transport across the BBB.

The model not only integrates the main processes that govern drug distribution into and within the brain, but also allows for the inclusion of parameters that are based on physiological data. We perform a sensitivity analysis to study the effect of a range of physiological drug-specific and system-specific parameters on the local concentration–time profiles of free and bound drug. Here, because the model is in 2D, we can distinguish between multidirectional processes (such as diffusion) and unidirectional processes (such as the brain ECF bulk flow). In addition, the square geometry of the model, in which the brain capillaries surround the brain ECF, enables the study of the local distribution of a drug. This combination of properties generates a model that is new in its form compared to earlier studies.

In the remaining parts of this article, we first explain the physiology on which our model is based in Sect. 2.1. In Sect. 2.2, we set up the model for drug transport through the brain ECF and drug binding. Then, we formulate the boundary conditions for drug transport across the BBB in Sect. 2.3. The values and units of the variables we use in our model are given in Sect. 2.4. In Sect. 3.1, we first assess the effect of both specific and non-specific binding in our model. Then, we study the effect of changing parameters, such as drug binding kinetics and BBB permeability, on drug concentration in Sects. 3.2 and 3.3. Finally, in Sect. 3.4, we use our model to show variations in drug concentration over space. We discuss and conclude our work in Sect. 4.

## The 2D Brain Tissue Unit

The purpose of our model is to describe the local concentrations of free and bound drug within the brain after the BBB. To that end, we formulate a model using the basic characteristics of a typical (2D) piece of brain tissue that is surrounded by capillaries (where the BBB is located). This is the brain tissue unit. We base our model on physiological values and choose the size and parameters in the model to correspond to the rat brain as for this, most data are available. As our model uses known physiological parameters, it can easily be translated to other species, including humans, by setting the parameters to values that match those of the species of interest.

In the 2D brain tissue unit, the brain capillaries surround the brain ECF. Here, drug exchanges between the blood plasma and the brain ECF by crossing the BBB and distributes within the brain ECF. In the rat brain, the distance between the capillaries is on average only $50 \, \upmu \hbox {m}$ (Jucker et al. 1990; Schlageter et al. 1999; Pardridge 2005; Tata and Anderson 2002). As the capillaries are widely distributed within the brain, many of these units eventually build up to the entire brain.

### Formulating the Model Based on the Physiology of the Brain

We aim for a model that covers all essential aspects of drug distribution within the brain: drug exchange between the blood plasma and the brain ECF (BBB transport), drug transport through the brain ECF by diffusion and brain ECF bulk flow and the kinetics of drug binding to specific and non-specific binding sites. Moreover, we aim for a model that represents the actual physiological geometry of the brain tissue unit, in which the brain capillaries surround the brain ECF.

**Fig. 1:**
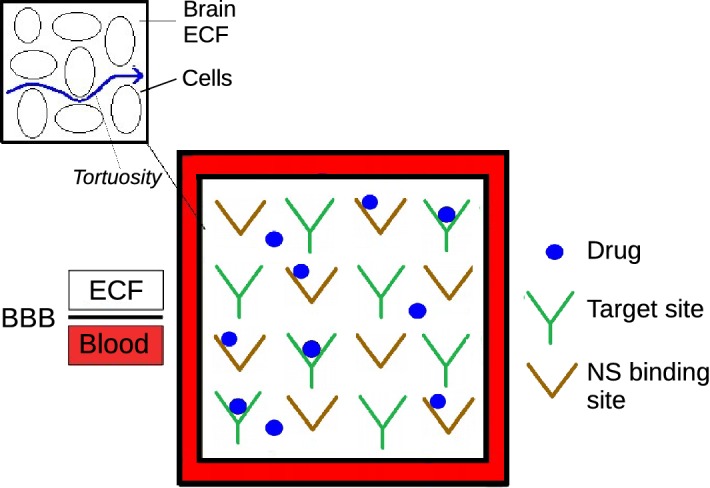
Sketch of one 2D brain tissue unit. Free drug exchanges between the blood plasma in the brain capillaries and the brain ECF by crossing the BBB, located at the level of the brain capillaries. Free drug distributes within the brain ECF and binds to both specific (target) and non-specific (NS) binding sites

We assume that the brain capillaries form square regions around the brain tissue unit, which contains the brain ECF. The unit is a square, where $(\textit{x},\textit{y}) \in [0, x_{\mathrm {r}}] \times [0, y_{\mathrm {r}}]$, with (0,0) located in the lower left corner and ($x_{\mathrm {r}}, y_{\mathrm {r}}$) in the upper right corner and *x* is the horizontal variable and *y* the vertical variable. Here, $x_r$ and $y_r$ both represent the distance between the brain capillaries and are therefore chosen to be equal to $50 \, \upmu \hbox {m}$. The advantage of modelling the brain tissue unit as a square is that it enables the connection of units and thus the extension to a larger scale. In the 2D model representation, the brain capillaries entirely surround the brain ECF and hence the domain. A sketch of the model representation of the brain tissue unit is shown in Fig. 1.

Here, drug is exchanged between the blood plasma in the brain capillaries and the brain ECF in the unit. Within the brain ECF in the unit, drug is transported by diffusion and brain ECF bulk flow. For simplicity, we do not consider cells and assume that the entire volume space of the brain ECF is available for drug distribution. However, cells are implicitly implemented as the hindrance the cells would impose on the transport of a drug through the brain ECF is taken into account in a tortuosity term, see Fig. 1. In a future model, the units can be connected to generate a larger-scale model in which regional differences can be assessed.

The exchange of drug between the brain ECF and the blood plasma in the surrounding brain capillaries across the BBB is described by the permeability of the BBB. For simplicity, we assume that the transport over the BBB is passive and therefore driven by diffusion in both directions.

We model the transport of a drug through the brain ECF within a unit by diffusion and brain ECF bulk flow. Drug diffusion through the brain ECF is restricted by hindrances imposed by the cells or by substances in the brain ECF. As a result, the actual, or effective, diffusion is different from the normal diffusion. This can be modelled by the tortuosity (Nicholson and Phillips 1981; Nicholson 2001). The tortuosity is defined as $\lambda = \sqrt{\frac{D}{D*}}$, where *D* is the diffusion coefficient in a medium without hindrances (like in water) and D* the effective diffusion coefficient in the brain ECF. Hence, D* is given by $\frac{D}{{\lambda }^2}$. Tortuosity differs between drugs, and drugs that are able to cross the cell membranes and enter brain cells show a larger value of tortuosity (Nicholson et al. 2011).

The brain ECF bulk flow is directed from the left boundary of the unit towards the right boundary and is the result of a pressure gradient along the brain ECF.

The brain ECF contains specific and non-specific binding sites. We assume that the total concentration of specific and non-specific binding sites is constant and that the binding sites do not move and are evenly distributed over the brain ECF. In addition to this, we assume that non-specific binding sites are more abundant than specific binding sites. Only a limited concentration of specific binding sites is available to which drugs can bind. Moreover, we assume that drug binding is reversible and drugs associate with and dissociate from their binding sites. Finally, we assume that binding to specific binding sites is stronger than to non-specific binding sites, e.g. we assume that drugs associate more easily with specific binding sites than with non-specific binding sites, but dissociate less easily from specific binding sites than from non-specific binding sites.

### Modelling Drug Transport Through the Brain ECF

In this section, we present the equations that describe the change in the concentration of drug in the brain ECF, where we base this model on the physiology in Sect. 2.1. Drug in the brain ECF moves by diffusion and brain ECF bulk flow and binds to specific and non-specific binding sites in the brain ECF. We describe the change in drug concentration in the brain ECF over time, in (s), and space by drug movement and drug binding as follows:

1 $$\begin{aligned} \frac{\partial C_{\mathrm {ECF}}}{\partial \mathrm {t}} = \frac{D}{\lambda ^2} \nabla ^2 C_{\mathrm {ECF}} - v \nabla C_{\mathrm {ECF}} -f_{\mathrm {binding}}(C_{\mathrm {ECF}}). \end{aligned}$$

Here, we denote the concentration of free drug in the brain ECF by $C_{\mathrm {ECF}}$ (mol$\, \hbox {L}^{-1}$). Furthermore, *D* ($\hbox {m}^2\, \hbox {s}^{-1}$) is the diffusion coefficient of free drug, $\lambda$ (no unit) is the tortuosity, *v* ($\hbox {m}\, \hbox {s}^{-1}$) is the brain ECF bulk flow in the x direction and $f_{\mathrm {binding}}$ is a function that describes the binding of the drug to specific and non-specific binding sites. We formulate $f_{\mathrm {binding}}$ in Sect. 2.2.1.

Equation (1) is similar to the models on drug transport through the brain ECF as described by Nicholson (e.g. Nicholson 2001 and Syková and Nicholson 2008). Here, we capture the entry and elimination of drug into and from the brain ECF by transport across the BBB with our boundary conditions, as formulated in Sect. 2.3.

#### Drug Binding Kinetics

Next, we model the kinetics of drug binding to specific and non-specific binding sites. We denote the concentration of drug bound to specific binding sites by ${B}_\mathrm {1}$ ($\upmu \hbox {mol}\,\hbox {L}^{-1}$) and the concentration of drug bound to non-specific binding sites by ${B}_\mathrm {2}$ ($\upmu \hbox {mol}\,\hbox {L}^{-1}$). We denote the total concentration of specific and non-specific binding sites by ${B}_\mathrm {1}^{\mathrm {max}}$ and ${B}_\mathrm {2}^{\mathrm {max}}$ ($\upmu \hbox {mol}\, \hbox {L}^{-1}$), respectively. As the total concentration of bound drug can never exceed the concentration of binding sites, this is also the maximum concentration of bound drug. The concentration of free specific and non-specific binding sites is thus described by $B_\mathrm {1}^{\mathrm {max}}-B_\mathrm {1}$ and $B_\mathrm {2}^{\mathrm {max}}-B_\mathrm {2}$, respectively. We describe the drug association rate as the product of the drug association rate constant $k_{\mathrm {on}}$, the concentration of free drug $C_{\mathrm {ECF}}$ and the concentration of free binding sites ($B_{\mathrm {max}}-B$). The drug dissociation rate is described as the product of the drug dissociation rate constant and the concentration of bound drug-binding site complexes. The binding of drugs to specific and non-specific binding sites is captured by two ODEs that describe the change in concentration of bound drug over time. These equations replace the term $\hbox {f}_{\mathrm {binding}}$ in Eq. (1).

In this way, we obtain the following system of equations:

2 $$\begin{aligned} \begin{aligned} \frac{ \partial C_{\mathrm {ECF}}}{{\partial } t}&= \frac{D}{{\lambda }^2} \nabla ^2 C_{\mathrm {ECF}} - v \nabla C_{\mathrm {ECF}} -k_{\mathrm {1on}}C_{\mathrm {ECF}} (B_{\mathrm {1}}^{\mathrm {max}} - B_{\mathrm {1}}) + k_{\mathrm {1off}}B_{\mathrm {1}} \\&\quad - k_{\mathrm {2on}} C_{\mathrm {ECF}} (B_\mathrm {2}^{\mathrm {max}} - B_\mathrm {2}) + k_\mathrm {{2off}}B_\mathrm {2}, \\ \frac{{\partial } B_\mathrm {1}}{{\partial } t}&= k_{\mathrm {1on}}C_{\mathrm {ECF}} (B_\mathrm {1}^{\mathrm {max}} - B_\mathrm {1}) - k_{\mathrm {1off}}B_\mathrm {1}, \\\ \frac{{\partial } B_\mathrm {2}}{{\partial } t}&= k_{\mathrm {2on}}C_{\mathrm {ECF}} (B_\mathrm {2}^{\mathrm {max}} - B_{\mathrm {2}}) - k_{\mathrm {2off}}B_\mathrm {2}, \\\ \end{aligned} \end{aligned}$$

where $k_{\mathrm {1on}}$ (($\upmu \hbox {mol}\, \hbox {L}^{-1}\,\mathrm {s)}^{-1}$) is the association rate constant for specific binding, $k_{\mathrm {1off}}$ ($\hbox {s}^{-1}$) is the dissociation rate constant for specific binding, ${k}_{\mathrm {2on}}$ [(($\upmu \hbox {mol}\, \hbox {L}^{-1}\,\mathrm {s)}^{-1}$] is the association rate constant for non-specific binding and $k_{\mathrm {2off}}$ ($\hbox {s}^{-1}$) is the dissociation rate constant for non-specific binding.

Initially, we assume that no drug is present in the brain ECF, hence

3 $$\begin{aligned} C_{\mathrm {ECF}}(x,y,t=0)=0, \end{aligned}$$

and hence, we also have that

4 $$\begin{aligned} B_{\mathrm {i}} (x,y,t=0) = 0, i=1,2. \end{aligned}$$

### Modelling Drug Transport Across the BBB

We explicitly model drug transport across the BBB with our boundary conditions. At the boundaries of the brain tissue unit, drug enters and exits the brain ECF from and to the blood plasma by crossing the BBB. There, a flux *J* ($\upmu \hbox {mol}\, \hbox {m}^{-2}\,\mathrm {s}^{-1}$) describes the amount of drug transported across the BBB per area per time. This flux results from the concentration difference between the blood plasma and the brain ECF and the permeability of the BBB and is described by

$$\begin{aligned} J=P(C_{\mathrm {pl}}-C_{\mathrm {ECF}}), \end{aligned}$$

where the permeability is denoted by *P* ($\hbox {m}\, \hbox {s}^{-1}$) and the concentration of drug in the blood plasma by $C_{\mathrm {pl}}$ ($\upmu \hbox {mol}\, \hbox {L}^{-1}$). On the other hand, this flux is proportional to the concentration gradient between the blood plasma and the brain ECF with the effective diffusion coefficient D* ($\hbox {m}^2\,\mathrm {s}^{-1}$) as proportionality constant, leading to

5 $$\begin{aligned} J = -D^* \frac{{\partial }{C_{\mathrm {ECF}}}}{{\partial } x} . \end{aligned}$$

Based on the fact that these fluxes should match, we find the following boundary conditions:

6 $$\begin{aligned} -D^* \frac{{\partial }{C_{\mathrm {ECF}}}}{{\partial } x} = P (C_{\mathrm {pl}} - C_{\mathrm {ECF}}), \end{aligned}$$

for $x=0$ and $y=0$, and

7 $$\begin{aligned} D^* \frac{ {\partial }{C_{\mathrm {ECF}}}}{{\partial } y} = P (C_{\mathrm {pl}} - C_{\mathrm {ECF}}) , \end{aligned}$$

for $$\textit{x}= x_{\mathrm {r}}$$ and $$\textit{y}= y _{\mathrm {r}}$$.

As mentioned before, we assume that *P* is a measure of passive transport across the BBB only. Moreover, we assume that the transport across the BBB is limited by the BBB permeability only, and not by the blood flow in the brain capillaries, which may be important for drugs that easily cross the BBB. We have chosen to omit this in this proof-of-concept 2D model, but in a more refined 3D model, more detailed descriptions of BBB transport can be taken into account.

For the concentration of drug in the blood plasma, $C_{\mathrm {pl}}$, which is time dependent, different descriptions exist, depending on the route of administration. A drug that is administered intravenously is modelled with the function:

8 $$\begin{aligned} C_{\mathrm {pl}} = C_0 e^{-k_e t} \end{aligned}$$

with

9 $$\begin{aligned} C_0 = \frac{Dose}{V_D}, \end{aligned}$$

see Rowland and Tozer (2005). Here, $C_0$ ($\upmu \hbox {mol}\, \hbox {L}^{-1}$) is the concentration of drug in the blood plasma at $t=0$, *Dose* ($\upmu \hbox {mol}$) is the molar amount of administered drug, $V_\mathrm {d}$ (L) is the distribution volume, which is the theoretical volume that is needed to contain the total amount of drug at the same concentration as in blood plasma, and $k_\mathrm {e}$ ($\hbox {s}^{-1}$) is the rate constant of elimination.

Similarly, the following function is used for a drug that is administered orally:

10 $$\begin{aligned} C_{\mathrm {pl}} = \frac{F{\cdot }Dose{\cdot } k_\mathrm {a}}{V(k_\mathrm {a}-k_\mathrm {e})} (e^{-k_\mathrm {e} t}-e^{-k_\mathrm {a} t}), \end{aligned}$$

see Rowland and Tozer (2005). Here, *F* (ratio from 0 to 1) is the bioavailability of the drug and $k_\mathrm {a}$ ($\hbox {s}^{-1}$) is the rate constant of absorption. Typically, $C_{\mathrm {pl}}$ of orally absorbed drug shows an initial increase that reflects drug absorption into the blood plasma and a subsequent decrease that reflects drug elimination from the blood plasma. We assume that $C_{\mathrm {pl}}$ is independent of $C_{\mathrm {ECF}}$, whereas in reality drug flows back into the blood plasma from the brain ECF. However, it has been reported that as the brain compartment is only a small part of the entire body, the small concentration of drug returning from the brain ECF back into the blood plasma does not affect the blood plasma kinetics (Sheiner et al. 1979; Hammarlund-Udenaes et al. 1997). In this paper, we investigate the local drug distribution within the 2D brain tissue unit for blood plasma profiles that result from oral administration and thus describe $C_{\mathrm {pl}}$ by expression (10).

### Model Values and Units

In Table 2, we give the range of values between which the quantities and parameters in our model can vary. These ranges are based on physiological values that are taken from studies in literature, where measurements and experiments are performed. References for these studies are also given in the table. Using a physiological range of values allows us to perform a sensitivity analysis and examine the effect of parameter values at both extremes of the physiological range on the behaviour of the model. As no experimental data are available on the kinetics of drug binding to non-specific binding sites, no data are given for $B_{\mathrm {2}}^{\mathrm {max}}$, $k_{\mathrm {2on}}$ and $k_{\mathrm {2off}}$. We will come back to this in the next section (Sect. 3).

**Table 2:** The parameters and units of the 2D brain tissue unit model

Parameter	Unit	Range of values	References
Effective diffusion coefficient $(\textit{D*})^\mathrm{a}$	$\mathrm {m}^2\,\mathrm {s} ^{-1}$	$10^{-11}$–$10^{-10}$	Nicholson et al. (2000)
			Nicholson et al. (2011)
Brain ECF bulk flow velocity (*v*)	$\hbox {m}\, \hbox {s}^{-1}$	$5 \times 10^{-8}$–$5\times 10^{-6}$	Saltzman (2000)
			Hladky and Barrand (2014)
BBB permeability $(\textit{P})^\mathrm{b}$	$\hbox {m} \, \hbox {s}^{-1}$	$10^{-10}$–$10^{-5}$	Wong et al. (2013)
Total concentration targets ($B_\mathrm {1}^{\mathrm {max}}$)	$\upmu \hbox {mol}\, \hbox {L}^{-1}$	$1 \times 10^{-3}$–$5\times 10^{-1}$	de Witte et al. (2016)
Target association constant ($k_{\mathrm {1on}}$)	($\upmu \hbox {mol} \, \hbox {L}^{-1} \,\hbox {s})^{-1}$	$10^{-4}$–$10^3$	de Witte et al. (2016)
Target dissociation constant ($k_{{\mathrm {1off}}}$)	$\hbox {s}^{-1}$	$10^{-6}$–$10^{1}$	de Witte et al. (2016)
Bioavailability (*F*)	–	0–1	Rowland and Tozer (2005)
Dose	$\upmu \hbox {mol}$	$10^{-1}$–$5 \times 10^3$	Rowland and Tozer (2005)
Absorption rate constant ($k_{\mathrm {a}}$)	$\hbox {s}^{-1}$	0–$2 \times 10^{-3}$	Rowland and Tozer (2005)
Elimination rate constant ($k_{\mathrm {e}}$)	$\hbox {s}^{-1}$	$10^{-1}$–$5 \times 10^{-3}$	Rowland and Tozer (2005)
Distribution volume (*V*)	L	0.01–$50 \times 10^{3}$	Rowland and Tozer (2005)

The physiological range of values of the parameters is given. These are based on references from the literature

$^\mathrm{a}$This equals $\frac{D}{\lambda ^2}$, see Nicholson et al. (2000, 2011)

$^\mathrm{b}$This is the range of values of *P* measured in both 2D and 3D assays. Typical values of *P* measured in 2D assays are within the range of $10^{-9}$–$10^{-7} \,\hbox {m}\, \hbox {s}^{-1}$ (Summerfield et al. 2007; Wong et al. 2013)

## Model Results

Before simulating the system of equations numerically, we have non-dimensionalised it and give the details in Appendix I. There, the spatial variables are scaled by the dimensions of a 2D brain tissue unit (50 by $50 \, \upmu \hbox {m}$) and the other variables and parameters with a characteristic scale. Next, the non-dimensionalised PDEs are spatially discretised where we use a well-established numerical procedure based on finite element approximations (Schiesser and Griffiths 2009). During the simulations, we use, unless otherwise indicated, a fixed set of parameter values, which is given in Table 3. We have chosen values that are within the physiological ranges given in Table 2.

We assume that there is oral administration and take $C_{\mathrm {pl}}$ the same in all the simulations, calculated as a time-dependent function (expression 10) and with the coefficients chosen as in Table 2.

**Table 3:** Model parameter values

Parameter	Unit	Value
D*	$\mathrm {m^2\,\mathrm {s}^{-1}}$	$5 \times 10^{-11}$
*v*	$\hbox {m}\, \mathrm {s^{-1}}$	$5 \times 10^{-7}$
*P*	$\hbox {m}\,\mathrm {s^{-1}}$	$10^{-9}$
$B_\mathrm {1}^{\mathrm {max}}$	${\upmu } \hbox {mol}\, \hbox {L}^{-1}$	$5 \times 10^{-2}$
$k_{\mathrm {1on}}$	$(\upmu \hbox {mol}\, \hbox {L}^{-1}\,\mathrm {s})^{-1}$	1
$k_{\mathrm {1off}}$	$\mathrm {s^{-1}}$	$10^{-2}$
$B_\mathrm {2}^{\mathrm {max}}$	${\upmu } \hbox {mol}\, \hbox {L}^{-1}$	50
$k_{\mathrm {2on}}$	$(\upmu \hbox {mol}\, \hbox {L}^{-1}\,\mathrm {s})^{-1}$	$10^{-2}$
$k_{\mathrm {2off}}$	$\mathrm {s^{-1}}$	1
*F*	–	1
Dose	${\upmu } \hbox {mol}$	30
$k_a$	$\mathrm {s^{-1}}$	$2 \times 10^{-4}$
$k_e$	$\mathrm {s^{-1}}$	$5 \times 10^{-5}$
*V*	L	20

The value of the default choice of the parameters is given together with their unit. The magnitude of these values is chosen to be within the physiological ranges given in Table 1

The literature lacks values of the parameters related to non-specific binding kinetics, e.g. the association and dissociation rates of drug binding to non-specific binding sites. Therefore, for now, we need to base the choices of these values on assumptions. First, we assume that drugs associate with non-specific binding sites less strongly, while they dissociate more easily. More specifically, we base the choice of $k_{\mathrm {2on}}$ and $k_{\mathrm {2off}}$ on modelling studies by McGinty and Pontrelli (2016) and Tzafriri et al. (2012) and take $k_{\mathrm {2on}}$ a factor 100 lower than $k_{\mathrm {1on}}$ and $k_{\mathrm {2off}}$ a factor 100 higher than $k_{\mathrm {1off}}$. In addition, as the concentration of drug is expected to be lower in the brain than in the arterial wall (as modelled in McGinty and Pontrelli 2016) because of the BBB, we expect relatively more non-specific binding sites in the brain ECF than in the arterial wall. Therefore, we choose $B_{\mathrm {2}}^{\mathrm {max}}$ to be a factor 1000 higher than $B_{\mathrm {1}}^{\mathrm {max}}$, which is higher than the factor 100 used by Tzafriri et al. (2012) and McGinty and Pontrelli (2016).

In the next Sects. (3.1–3.4), we give the concentration–time profiles as well as the local drug distributions of free and bound drug within the brain ECF in the single brain tissue unit. In the concentration–time profiles, the concentration is given on a log scale versus time. Moreover, we have chosen to plot the concentrations in one point in the (*x*, *y*)-domain, which is located in the middle of the unit. On longer time scales and with the set of parameter values we choose (Table 2), we find that after an initial difference the concentration–time profiles would look approximately the same in any other point of the (*x*, *y*)-domain. This can be seen in the local drug distribution plots (Figs. 8, 9, 10) given in the entire (*x*, *y*)-domain of the brain tissue unit for various times in Sect. 3.4. In all of the plots, we use the colour codes red, blue, green and brown for $C_{\mathrm {pl}}$, $C_{\mathrm {ECF}}$, $B_1$ and $B_2$, respectively. In the next sections, we show the influence of several physiological parameters, related to binding kinetics and permeability, on the concentration–time profiles of $C_{\mathrm {ECF}}$, $B_1$ and $B_2$.

### The Effect of Drug Binding on the Concentration–Time Profiles of Drug in the Brain ECF

**Fig. 2:**
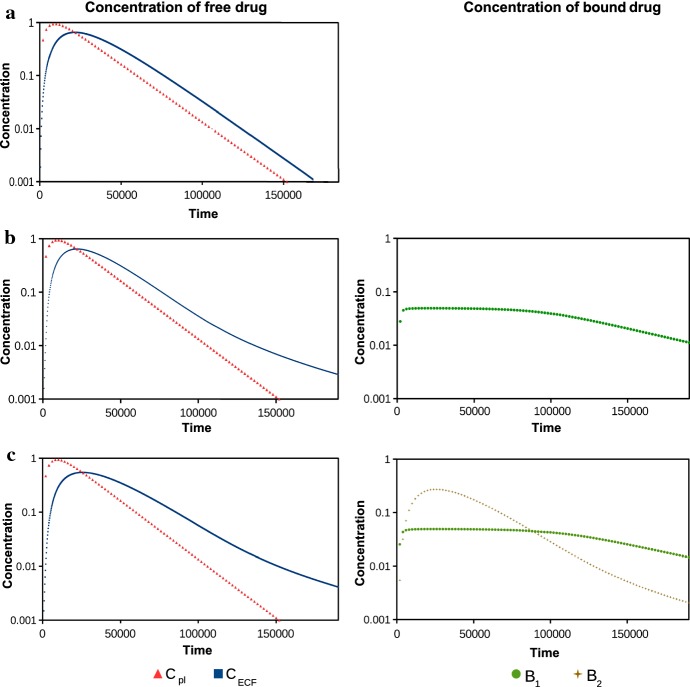
The concentration–time profiles in log scale of the drug in the blood plasma ($C_{\mathrm {pl}}$) and in the brain ECF ($C_{\mathrm {ECF}}$) on the left and of drug bound to its target sites ($B_1$) and non-specific binding sites ($B_2$) on the right. In **a**, we plot the concentration of free drug without binding, in **b** with specific binding and in **c** with both specific and non-specific binding. Parameters are as in Table 3

To investigate the effect of drug binding on the concentration of free drug within the brain ECF, we plot the concentration–time profile of free drug within the brain ECF, $C_{\mathrm {ECF}}$, with and without the presence of binding sites. The concentration–time profile of $C_{\mathrm {ECF}}$ without binding is shown in Fig. 2a (left), together with the concentration–time profile of $C_{\mathrm {pl}}$. Here, we find that the concentration–time profile of $C_{\mathrm {ECF}}$ follows that of $C_{\mathrm {pl}}$ with a delay. Moreover, we find that $C_{\mathrm {ECF}}$ is lower than $C_{\mathrm {pl}}$ before and at its peak concentration, but after that, $C_{\mathrm {ECF}}$ is higher than $C_{\mathrm {pl}}$. This reflects that here, free drug not only slowly enters the brain ECF, but also slowly leaves the brain ECF, due to a low permeability of the BBB.

The concentration–time profile of $C_{\mathrm {ECF}}$ in the presence of specific binding sites is shown in Fig. 2b (left). In Fig. 2b (right), we show the concentration of drug bound to specific binding sites, $B_1$. When we compare the concentration–time profiles of $C_{\mathrm {ECF}}$ in Fig. 2a (left) and Fig. 2b (left), we observe that the decrease in $C_{\mathrm {ECF}}$ towards the end of the simulation is slowed down in the presence of specific binding sites. Figure 2b (right) shows that $B_1$ quickly reaches a maximum. The reason for this is that free drug strongly associates with the limited concentration of specific binding sites. Meanwhile, drug dissociates slowly, which is reflected by a slow decrease in $B_1$. This decrease in $B_1$ follows the decrease in $C_{\mathrm {ECF}}$ and is caused by the release of drug from the specific binding sites.

The decrease in $C_{\mathrm {ECF}}$ after its peak is even stronger in the presence of non-specific binding sites in addition to specific binding sites (Fig. 2c (left)). The concentration–time profile of $B_2$ greatly resembles that of $C_{\mathrm {ECF}}$ (Fig. 2c (right)). This is thought to be caused by the combination of a high concentration of non-specific binding sites and a fast dissociation of the drug. Due to these factors, $B_{\mathrm {2}}^{\mathrm {max}}$ exceeds the concentration of free drug. Thus, the concentration of the free non-specific binding sites is always sufficiently high for free drugs to bind to. Therefore, the concentration–time profile of $B_2$ is proportional to that of $C_\mathrm {ECF}$. Note that all concentrations will eventually decay to zero when we run the simulation for a longer time since $C_{\mathrm {pl}}$ decays to zero.

**Fig. 3:**
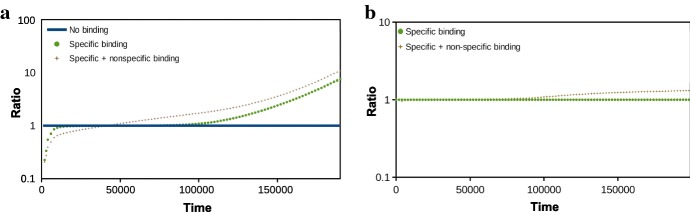
Concentration ratios over time of free and bound drug in the brain ECF in log scale. In **a**, the concentration ratios of $C_{\mathrm {ECF}}$ with binding (the ratio of $C_{\mathrm {ECF}}$ in the presence of only specific binding with respect to $C_{\mathrm {ECF}}$ without binding and the ratio of $C_{\mathrm {ECF}}$ in the presence of both specific and non-specific with respect to $C_{\mathrm {ECF}}$ without binding) are shown. In **b**, the concentration ratio over time of $B_1$ in the presence of specific and non-specific binding with respect to $B_1$ with only specific binding is shown

For clarity, we plot the same data on the concentration of free drug in the brain ECF in Fig. 3a as the ratio of $C_{\mathrm {ECF}}$ with binding and $C_{\mathrm {ECF}}$ without binding. Here, we see that $C_{\mathrm {ECF}}$ in the presence of binding is initially lower but later in time higher compared to when no binding is present. This effect is mainly due to specific binding; the inclusion of non-specific binding enhances the effect only slightly. In Fig. 3b, we plot the ratio of $B_1$ with and without non-specific binding. There, we see that in the presence of non-specific binding, $B_1$ slightly increases at the end of the simulation compared to when non-specific binding is not included.

**Fig. 4:**
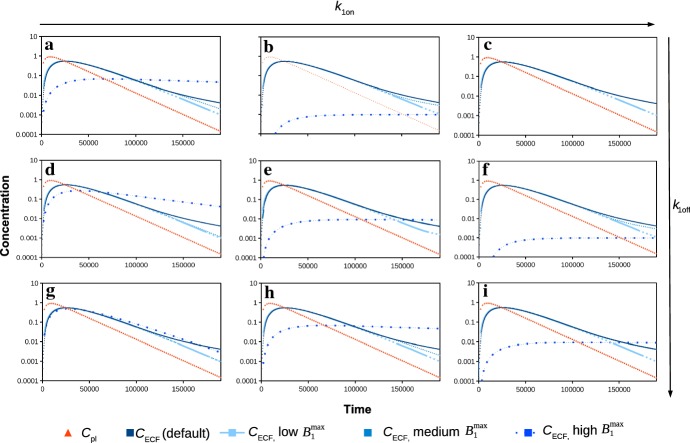
Concentration–time profiles on a log scale of $C_{\mathrm {ECF}}$ for various parameters in comparison with $C_{\mathrm {ECF}}$ for the default parameter set and of $C_{\mathrm {pl}}$. Here, $k_{\mathrm {1on}}$ is varied from 0.01 (left) to 1 (middle) and 10 (right) times the default value and $k_{\mathrm {1off}}$ is varied from 0.1 (top) to 1 (middle) and 10 (bottom) times the default value. In all of the graphs, $B_{\mathrm {1}}^{\mathrm {max}}$ is varied from 0.01 (low) to 1 (medium) and 100 (high) times the default value

### The Effect of the Kinetics of Drug Binding to Specific Binding Sites on Drug Concentrations Within the Brain ECF

Next, we study the influence of the various parameters related to the kinetics of specific binding on $C_{\mathrm {ECF}}$. We investigate combinations of several values of $k_{\mathrm {1on}}$, $k_{\mathrm {1off}}$ and $B_{\mathrm {1}}^{\mathrm {max}}$. In Fig. 4, the log concentration–time profiles of $C_{\mathrm {ECF}}$ are shown in nine sub-figures for several combinations of the values of $k_{\mathrm {1on}}$ and $k_{\mathrm {1off}}$. In the figure, $k_{\mathrm {1on}}$ increases from left to right and $k_{\mathrm {1off}}$ increases from top to bottom. Additionally, $B_{\mathrm {1}}^{\mathrm {max}}$ is varied, and therefore, three different graphs for $C_{\mathrm {ECF}}$ are shown in each sub-figure, together with $C_{\mathrm {ECF}}$ for the default parameters and $C_{\mathrm {pl}}$. The values of these parameters are changed as follows: $B_{\mathrm {1}}^{\mathrm {max}}$ and $k_{\mathrm {1on}}$ are varied from 0.01, 1 and 10 times the default value (Table 2) and $k_{\mathrm {1off}}$ is varied from 0.1, 1 and 10 times the default value (Table 2).

We observe that changing the association and dissociation rate constants $k_{\mathrm {1on}}$ and $k_{\mathrm {1off}}$ affects the decrease in $C_{\mathrm {ECF}}$ after its peak, see Fig. 4. In addition, for a larger $k_{\mathrm {1on}}$, drugs associate faster with their target sites, which can be seen by a decrease in $C_{\mathrm {ECF}}$. Moreover, with increasing $k_{\mathrm {1off}}$, drugs dissociate faster, which is visible as an increase in $C_{\mathrm {ECF}}$. This effect is most prominent for a higher value of $B_{\mathrm {1}}^{\mathrm {max}}$. In addition, when $k_{\mathrm {1on}}$ is lower and $k_{\mathrm {1off}}$ is higher (0.01 and 10 times the default value, respectively), $C_{\mathrm {ECF}}$ decreases more quickly after the peak than when $k_{\mathrm {1on}}$ is higher or when $k_{\mathrm {1off}}$ is smaller (Fig. 4g). Again, these effects are mainly visible for a larger $B_{\mathrm {1}}^{\mathrm {max}}$. This shows the relevance of looking at a combination of parameter values instead of varying just one parameter. Finally, we observe that increasing $B_{\mathrm {1}}^{\mathrm {max}}$ strongly lowers the peak concentration of $C_{\mathrm {ECF}}$ as well as the downward slope after the peak (Fig. 4a, b, e, h, i).

**Fig. 5:**
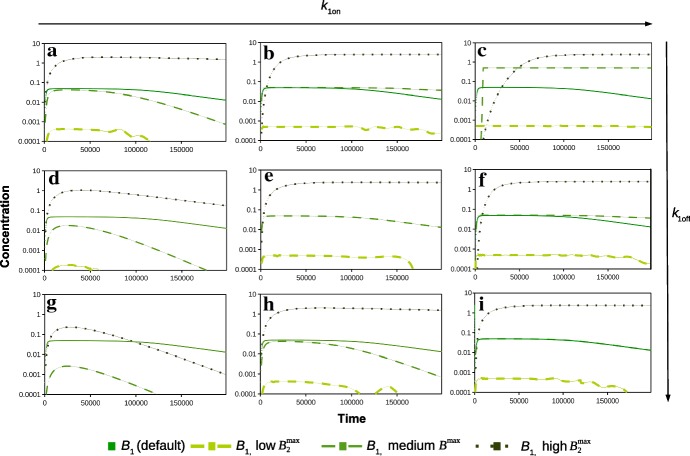
Concentration–time profiles on a log scale of $B_{1}$ for various parameters in comparison with the log concentration–time profiles of $B_{1}$ with the default parameter set. Again, $k_{\mathrm {1on}}$ is varied from 0.01 (left) to 1 (middle) and 10 times the default value (right) and $k_{\mathrm {1off}}$ is varied from 0.1 (top) to 1 (middle) and 10 (bottom) times the default value. In all of the graphs, $B_{\mathrm {1}}^{\mathrm {max}}$ is varied from 0.01 (low) to 1 (medium) and 100 (high) times the default value

**Fig. 6:**
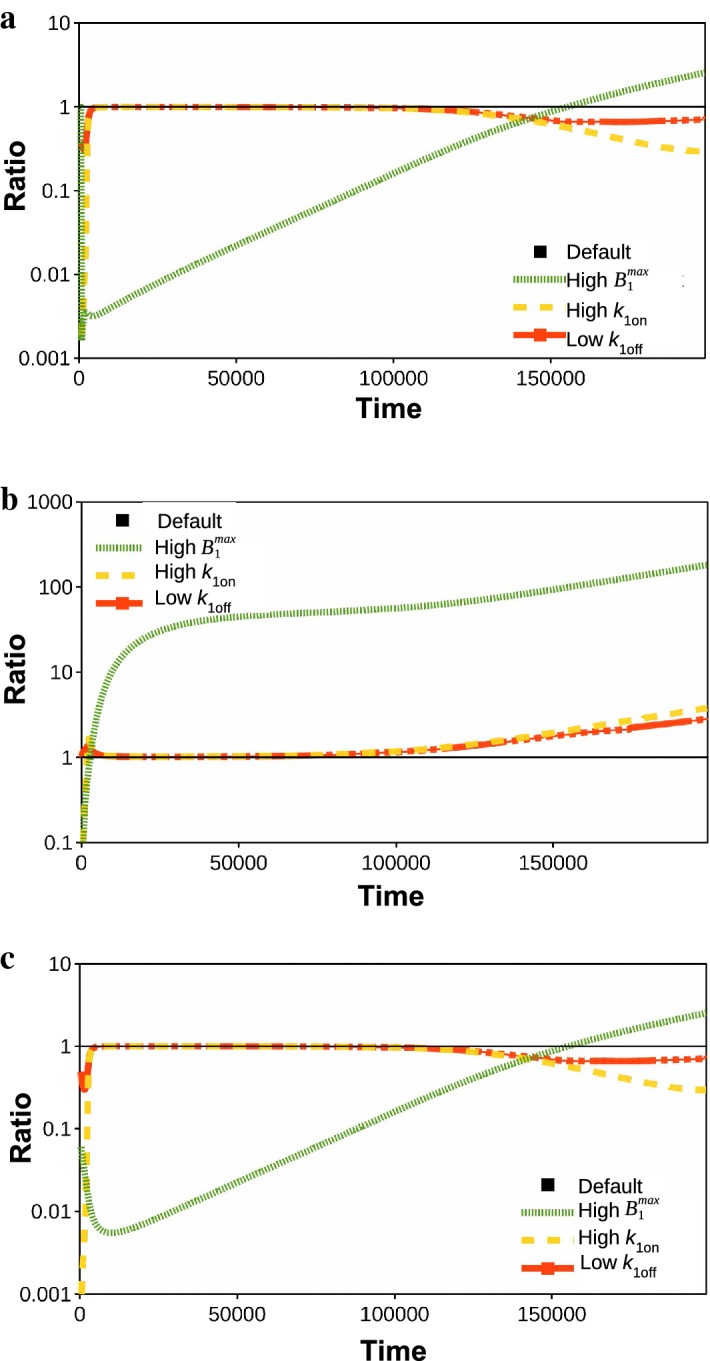
Concentration ratios over time of free and bound drug in the brain ECF on a log scale. The ratios of $C_{\mathrm {ECF}}$ (**a**), $B_{\mathrm {1}}$ (**b**) and $B_{\mathrm {2}}$ (**c**) with altered specific binding parameters (high $B_{\mathrm {1}}^{\mathrm {max}}$ of 100 times the default, high $k_{\mathrm {1on}}$ of 100 times the default and decreased $k_{\mathrm {1off}}$ of 0.1 times the default) to drug concentration with default parameters are shown

We are also interested in the effects of $k_{\mathrm {1on}}$, $k_{\mathrm {1off}}$ and $B_{\mathrm {1}}^{\mathrm {max}}$ on the concentration–time profiles of bound drug, in particular those of drug bound to specific binding sites. Therefore, in Fig. 5 we use the same set of combinations of values for $k_{\mathrm {1on}}$, $k_{\mathrm {1off}}$ and $B_{\mathrm {1}}^{\mathrm {max}}$ to plot the log concentration–time profile of $B_1$. Figure 5 shows that for the low and default values of $B_{\mathrm {1}}^{\mathrm {max}}$, when $k_{\mathrm {1on}}$ is increased, $B_1$ increases faster to higher levels for the default $B_1$. Moreover, the decrease in $B_1$ is less strong. When we increase $k_{\mathrm {1off}}$, the peak concentrations of $B_1$ decrease, while $B_1$ decreases more quickly after the peak for a low and medium $k_{\mathrm {1on}}$. As $B_{\mathrm {1}}^{\mathrm {max}}$ represents the total concentration of specific binding sites, it is not surprising that an increased $B_{\mathrm {1}}^{\mathrm {max}}$ corresponds to an increased concentration of bound drug $B_1$. Increasing $B_{\mathrm {1}}^{\mathrm {max}}$ obviously increases $B_1$, but also mitigates the effects of a changed $k_{\mathrm {1on}}$ or $k_{\mathrm {1off}}$. Figure 5 shows that when $B_{\mathrm {1}}^{\mathrm {max}}$ is high, for most values of $k_{\mathrm {1on}}$ and $k_{\mathrm {1off}}$, $B_1$ stays close to its maximal value during most of the simulation.

The above observations are more clear when looking at the ratio of concentrations, as shown in Fig. 6. There, we vary one parameter different from the default set and then plot the ratio of drug concentration for the default parameter set and the new parameter set. In the different curves, we take $B_{\mathrm {1}}^{\mathrm {max}}$ and $k_{\mathrm {1on}}$ 100 times their default values and $k_{\mathrm {1off}}$ 0.1 its default value. Again, we observe that for a larger value of $B_{\mathrm {1}}^{\mathrm {max}}$, $C_{\mathrm {ECF}}$ strongly decreases during most of the simulation, although it does increase towards the end of the simulation when drug starts to dissociate from its target site, see Fig. 6a. Moreover, we observe once more that different values of $k_{\mathrm {1on}}$ and $k_{\mathrm {1off}}$ only moderately cause $C_{\mathrm {ECF}}$ to decrease towards the end of the simulation. In line with this, we see in Fig. 6b that for a high $B_{\mathrm {1}}^{\mathrm {max}}$, $B_\mathrm {1}$ strongly increases, whereas for a higher $k_{\mathrm {1on}}$ and for a smaller $k_{\mathrm {1off}}$, $B_\mathrm {1}$ increases only slightly towards the end of the simulation. Finally, we observe in Fig. 6c that the profile of $B_\mathrm {2}$ follows that of $C_{\mathrm {ECF}}$. This is explained by the high concentration of non-specific binding sites and weak binding, leading to non-saturable binding kinetics (see also Sect. 3.2).

### The Influence of Permeability on the Concentration Profiles of Drug in the Brain ECF

Next, we study how the BBB permeability, *P*, influences the drug concentrations within the brain ECF. Here, we have chosen the default value of *P* to be $P = 10^{-9}$ ($\hbox {m}\cdot \hbox {s}^{-1}$). We increased *P* to 10 times its default value and decreased *P* to 0.1 times its default value, which is the lowest physiological value of *P* (Table 1) and describes the permeability of drugs that have difficulties of passing the BBB. In contrast, a higher value of *P* corresponds to drugs that can more easily pass the BBB.

**Fig. 7:**
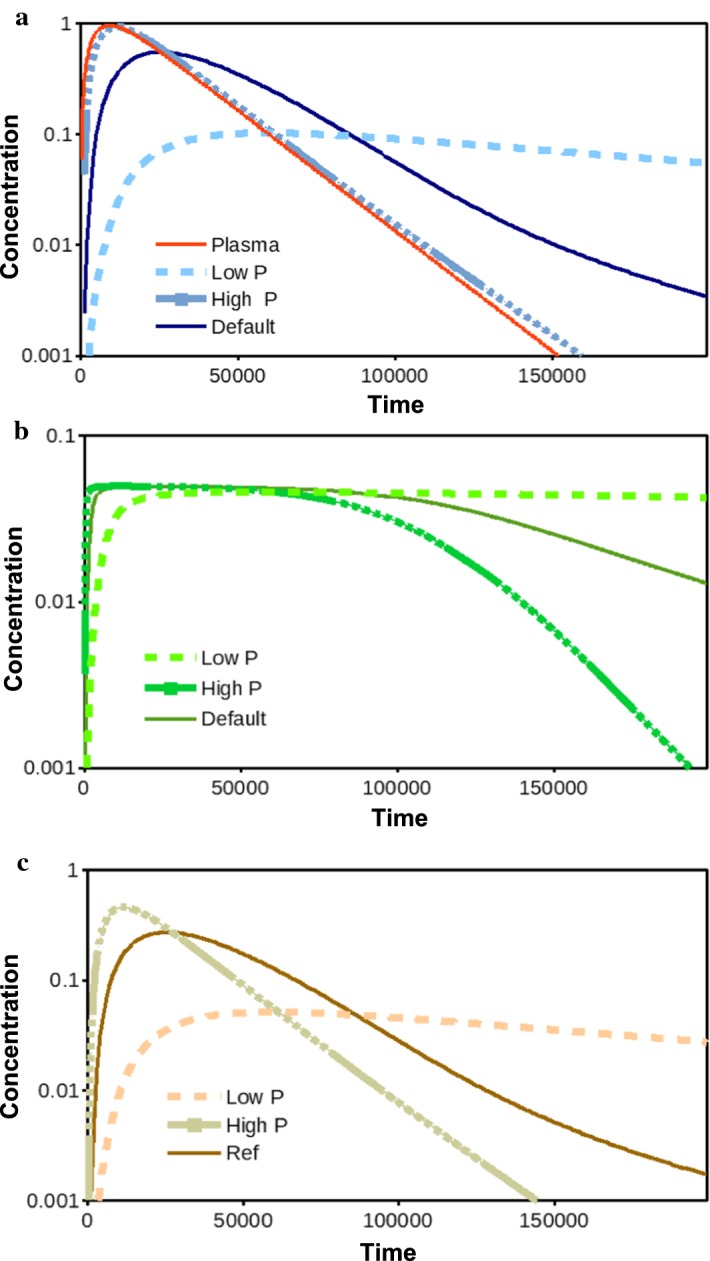
The effect of changing the permeability *P* on the log concentration–time profiles of $C_{\mathrm {ECF}}$, $B_1$ and $B_2$ is shown in **a**–**c**, respectively, for a low (0.1 times the default value) and high (10 times the default value) value of *P*. The concentration–time profiles of $C_{\mathrm {pl}}$ and $C_{\mathrm {ECF}}$ with the default value of *P* are shown as a reference in each sub-figure

In Fig. 7a, we plot $C_{\mathrm {ECF}}$ for the various choices of *P*, combined with $C_{\mathrm {pl}}$. We see that when *P* is larger, the concentration–time profile of $C_{\mathrm {ECF}}$ strongly follows that of $C_{\mathrm {pl}}$. Note that after this maximum, $C_{\mathrm {ECF}}$ lies slightly above $C_{\mathrm {pl}}$. For the smaller *P* of 0.1 times the default value, $C_{\mathrm {ECF}}$ increases and decreases more slowly than the default, as drug both enters and leaves the brain ECF more slowly. The peak concentration of $C_{\mathrm {ECF}}$ is also lower.

The BBB permeability *P* also influences the concentration–time profile of bound drug. In Fig. 7b, we plot $B_\mathrm {1}$ and observe that, for a higher value of *P*, $B_\mathrm {1}$ rapidly increases to a maximum. However, $B_\mathrm {1}$ starts to decrease quite fast again. The decrease in $B_\mathrm {1}$ starts when so much free drug has flown back through the more permeable BBB, that the concentration of free drug is not sufficiently high to bind to all of the free binding sites. In contrast, when *P* is lower, $B_\mathrm {1}$ increases more slowly and limits to a certain value. Only after a long time (longer than the simulation time), $B_\mathrm {1}$ decreases. In Fig. 7c, where $B_\mathrm {2}$ is given, it is shown that, when *P* is larger, $B_\mathrm {2}$ closely follows the concentration of $C_{\mathrm {ECF}}$, as given in Sect. 3.2. Again, this is due to the high concentration of the non-specific binding sites, $B_{\mathrm {2}}^{\mathrm {max}}$ and weak binding of drug to the non-specific binding sites. For a smaller *P*, $B_\mathrm {2}$, behaves like $C_{\mathrm {ECF}}$ and slowly increases to a smaller maximum value and then slowly decreases.

### The Local Drug Distribution Within the Brain Tissue Unit

**Fig. 8:**
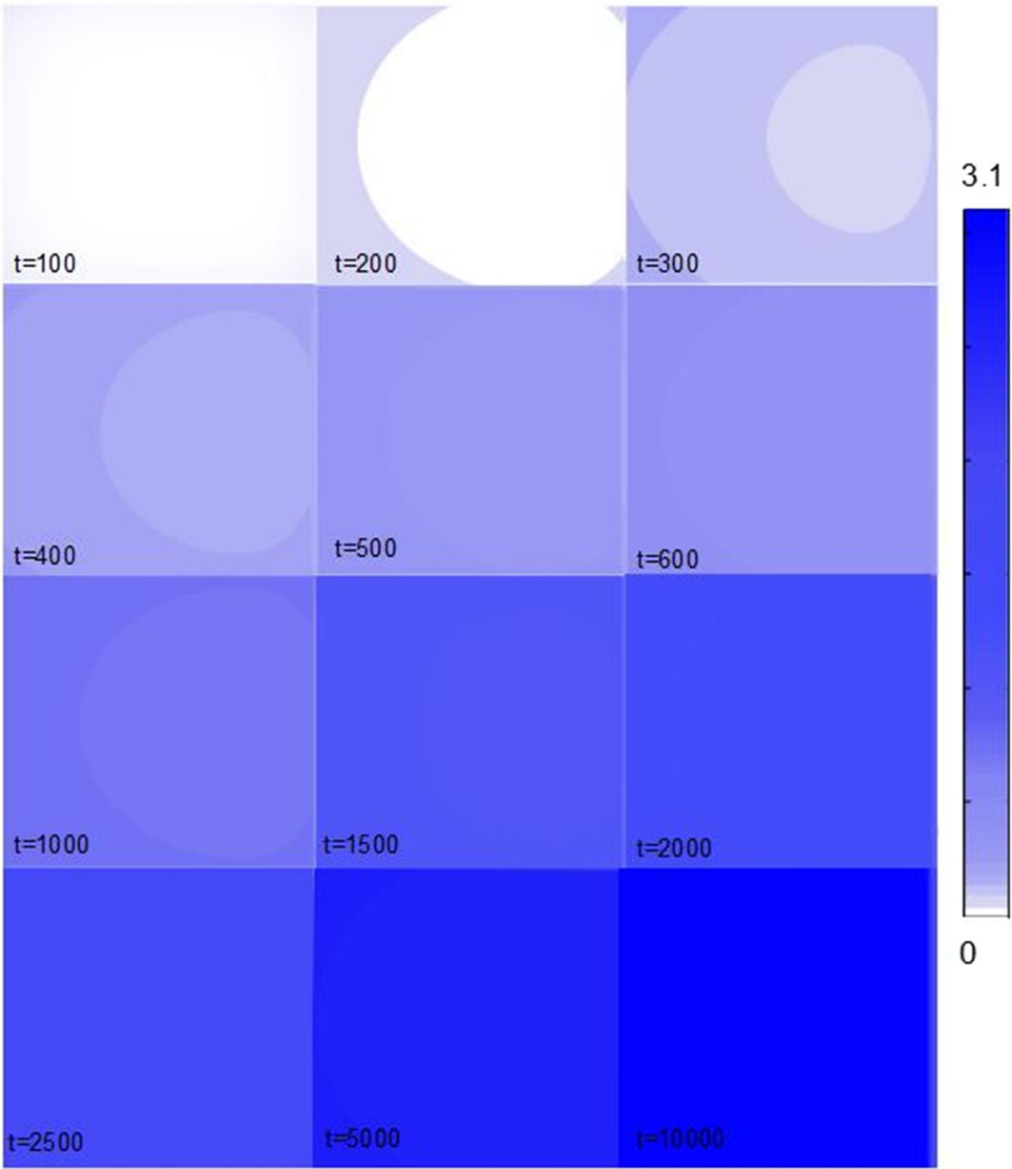
The local distribution of $C_{\mathrm {ECF}}$ within the 2D brain tissue unit is shown at several time steps. The concentration of the free drug is indicated by the shades of the colour bar, where darker colours correspond to higher concentrations (Color figure online)

In the previous section, we have shown the effects of drug binding and BBB permeability on the concentration–time profiles of a drug at one point of the brain tissue unit. A great advantage of our model is that it allows to study the local distribution of a drug within the entire brain tissue unit. In this section, we show that our model is able to detect local concentration differences in $C_{\mathrm {ECF}}$, $B_1$ and $B_2$ that arise within the domain. In these simulations, we choose several parameters to have different values from those in Table 2 for having a more extreme view on their impact on local drug distribution, as shown in Table 4. We show the concentrations at different times until the drug has distributed evenly over the unit. A time course of the local drug distribution within the unit shows that local concentration differences can be detected (Fig. 8). It can be seen that the concentrations rise most prominently on the boundaries of the brain tissue unit, where the BBB is located and drug flows in from the blood plasma in the brain capillaries. Moreover, the drug concentration is slightly higher at the left side of the unit than at the right side. This asymmetric distribution is a result of the unidirectional transport mediated by the brain ECF bulk flow. With time, the concentration differences become smaller due to diffusion, until the drug concentrations are evenly distributed over the brain tissue unit. Here, the time scale is very small, as also the area of distribution (one unit of 50 by $50 \, \upmu \hbox {m}$) is small.

**Fig. 9:**
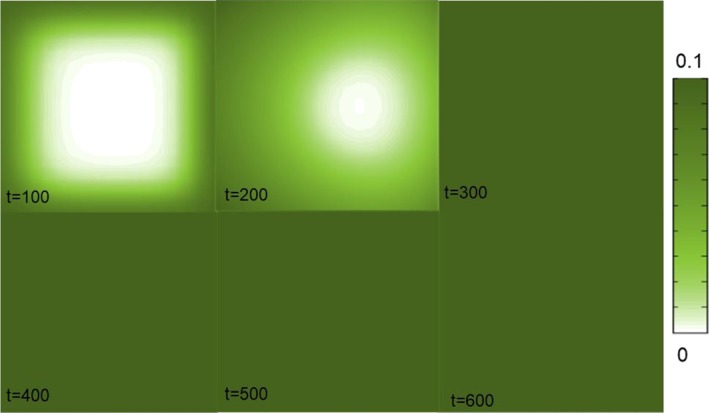
The local distribution of $B_{1}$ within the 2D brain tissue unit is shown at several time steps. Darker colours correspond to higher concentrations (Color figure online)

**Fig. 10:**
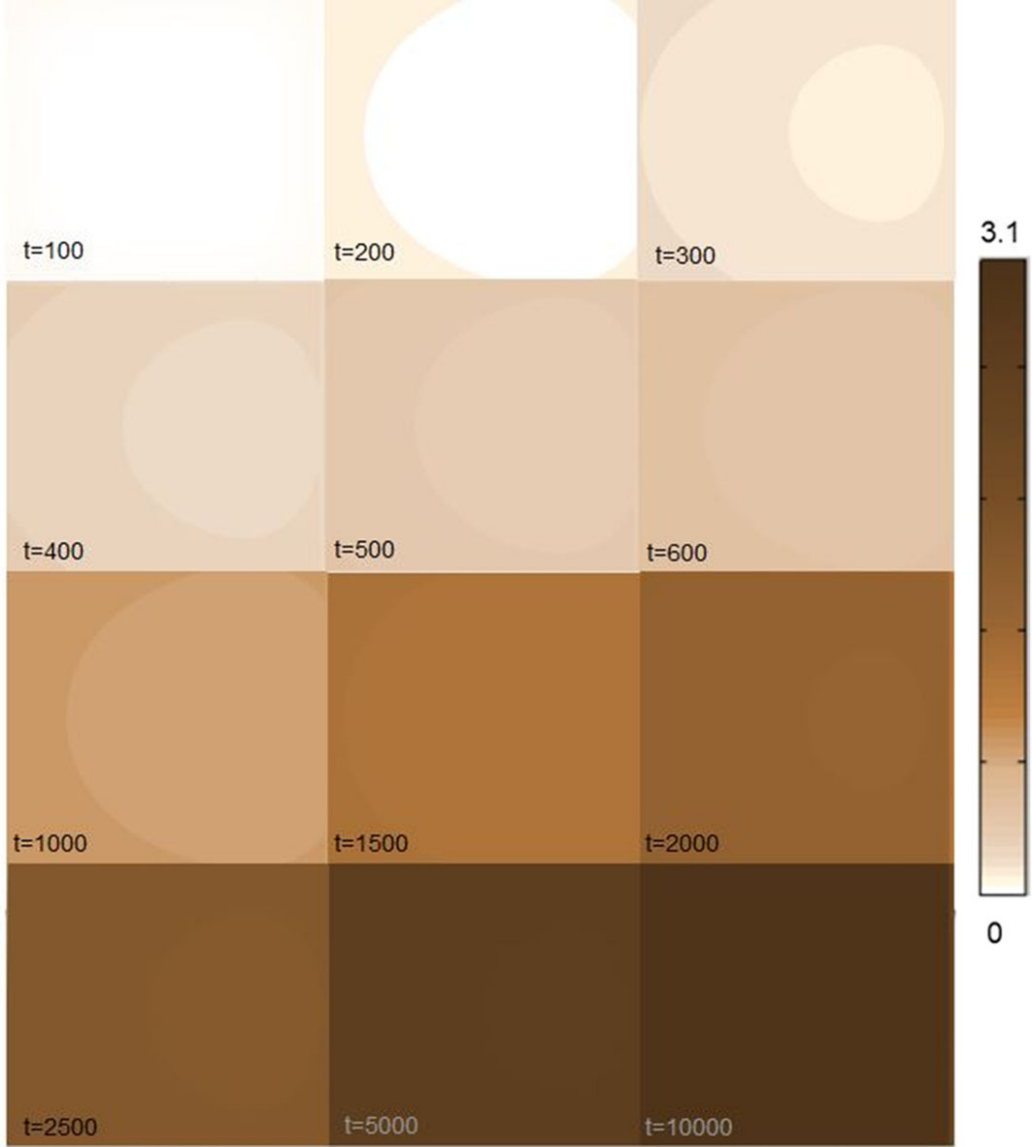
The local distribution of $B_{2}$ within the 2D brain tissue unit is shown for the same time steps as $C_{\mathrm {ECF}}$ in Fig. 8. A darker corresponds to a higher concentration (Color figure online)

In Fig. 9, we show the time course of changes in the local distribution of $B_1$. We observe that drug first binds to the specific binding sites at the boundaries of the unit nearby the BBB. At later times, the drug reaches the specific binding sites that are located in the middle of the unit and hence the concentration of $B_1$ increases there. Specific binding sites are quickly saturated as $k_{\mathrm {1on}}$ is large. This is shown by the concentration of drug bound to its target that quickly reaches its maximal value all over the unit. Finally, we give the local distribution of $B_2$ as a function of time in Fig. 10. There, we observe that the time course of the local distribution of $B_2$ is similar to that of $C_{\mathrm {ECF}}$. First, drug binds to the non-specific binding sites closer to the BBB at the boundaries of the unit and then reaches the non-specific binding sites in the middle of the unit.

**Table 4:** Model parameters

Quantity	Unit	Magnitude
$\textit{D*}$	$\hbox {m}^2 \,\mathrm {s}^{-1}$	$5 \times 10^{-12}$
Dose	$\upmu \hbox {mol}$	100
*P*	$\hbox {m} \, \hbox {s}^{-1}$	$10^{-7}$
$k_{\mathrm {1on}}$	$(\upmu \hbox {mol} \, \hbox {L}^{-1}\, \mathrm {s})^{-1}$	10
$B_{\mathrm {1}}^{\mathrm {max}}$	$\upmu \hbox {mol} \, \hbox {L}^{-1}$	$1 \times 10^{-1}$
$B_{\mathrm {2}}^{\mathrm {max}}$	$\upmu \hbox {mol} \, \hbox {L}^{-1}$	100

## Discussion

In this article, we have developed a mathematical model that describes a single 2D brain tissue unit. This unit represents the smallest building block of the brain and consists of surrounding blood capillaries, the BBB, the brain ECF and drug binding sites. The model enables us to integrate the processes that determine the local concentration–time profiles of a drug within the brain ECF, i.e. BBB permeability, drug transport and drug binding, and study their interdependence. With this 2D brain tissue unit model, we have aimed to improve current understanding of local drug distribution within the brain ECF.

The distribution of a drug within the brain ECF was previously described in mathematical models by Nicholson and Phillips (1981), Nicholson and Syková (1998), Nicholson (2001) and Syková and Nicholson (2008). However, they model drug transport through the brain ECF following drug administration directly into the brain. In our model, we describe the more common situation, where drug may enter the brain via cerebral blood, upon oral or venous administration of the drug. This means that the drug enters the brain via the capillary blood and distributes to the brain ECF via the BBB.

We have studied the influence of the BBB permeability on local drug concentration profiles within the brain. In disease conditions, BBB permeability parameter values may change and the understanding of their effect on local drug distribution within the brain has therefore a high clinical relevance, see, e.g. Hawkins and Davis (2005) and Zlokovic (2008) for excellent reviews on this topic. We have shown that with a higher permeability, or an increase in *P*, the concentration of free drug in the brain ECF, $C_\mathrm {ECF}$, is not only higher than under normal conditions, but also shows a different concentration–time profile, which more closely follows $C_{\mathrm {pl}}$ (Fig. 7).

After crossing the BBB, the drug distributes within the brain by diffusion and brain ECF bulk flow. Some research groups have argued that the brain ECF bulk flow velocity *v* has little influence on the distribution of a drug within the brain, especially on a short distance (Nicholson 2005; Syková and Nicholson 2008; Wolak and Thorne 2013). We have included brain ECF bulk flow in our model as there is evidence of the existence of bulk flow in the brain ECF (Cserr and Ostrach 1974; Abbott 2004) and that it may be a relevant means by which drugs distribute in the brain ECF (de Lange and Danhof 2002; Cserr and Ostrach 1974). Correspondingly we find in our model an effect of the brain ECF bulk flow velocity *v*, of which the value is within the reported physiological range (Table 2), on the distribution of drug within one brain tissue unit, see Figs. 8, 9 and 10.

Drug binding is of major importance for the effect of a drug. Therefore, as an extension of the model of Nicholson, we have added two ordinary differential equations to describe the change in concentration of drug bound to specific and non-specific binding sites. Herewith, we describe the interdependence of the concentrations of free and bound drug. We have shown that drug binding within the brain ECF makes the free drug concentrations in the 2D brain tissue unit rise and fall slower, see Fig. 2. Moreover, we have found that increasing $k_{\mathrm {1on}}$ lengthens the time period in which specific binding sites are saturated, i.e. when the concentration of drug bound to these specific binding sites, $B_1$, is at its maximal value (see Fig. 5). This corresponds to a recent study by de Witte et al. (2016), where it is stated that a high association rate constant can substantially reduce the rate of decline of target-occupancy.

In addition to specific binding, we have included non-specific binding in our model, because the concentration of drug bound to non-specific binding sites has implications for the concentration of free drug that is available to specific binding sites. A recent paper by McGinty and Pontrelli (2016) demonstrates the importance of modelling specific binding and non-specific binding as two different processes since they have other types of kinetics: specific binding sites are quickly saturated because of their low concentration and strong association with the drug, while non-specific binding sites are unlikely to become saturated due to their high capacity and weak binding. With the assumption that binding of drug to non-specific binding sites follows non-saturable binding kinetics, we have shown in Fig. 2c that non-specific binding indeed influences the concentration–time profile of free drug and that the concentration of non-specific binding sites is proportional to that of free drug.

In our model, we assume that all parameters are constant in time and space, whereas time-dependent and space-dependent changes in parameters are of course possible. We should, however, add that within a brain tissue unit as small as the one we model, local differences in properties, such as local differences in BBB permeability or concentrations of binding sites, are unlikely to be seen. For this reason, we have assumed that all parameter values are constant over space.

With our model, we have shown the local distribution of a drug within the 2D brain tissue unit. As the brain tissue unit is entirely surrounded by the brain capillaries, drugs within the brain tissue unit quickly distribute over the small area enclosed by the brain capillaries. We have shown that local concentration differences within the unit may exist, or, more specifically, that in the early time steps, differences in local drug concentrations could be seen.

On the basis of this 2D model, we are now able to extend the model to three dimensions. In 3D, the differences in local drug concentrations are expected to be more pronounced, since the brain capillaries are located only at the ribs of the cube, so that the point in the middle of the cube is further away from the capillaries compared to in 2D. This leads to a more realistic prediction of the concentration of drug that is present within the brain. Moreover, it allows for a comparison with experimental data from specific drugs, and therefore, it may provide insight into processes that are not known by experimental data, such as the kinetics of non-specific binding.

A 3D model allows for further refinement of the model. For example, as indicated, we have focused on passive transport across the cells of the BBB. However, drug can be actively transported into or out of the brain, mediated by, depending on the size of the drug, specific carriers or efflux transporters (Strazielle and Ghersi-Egea 2013). This can strongly influence the concentration of free drug in the brain (Hammarlund-Udenaes et al. 1997; De Boer et al. 2003). Moreover, drug may also be transported through the space between the cells (paracellular transport). This is particularly important for small, hydrophilic drugs or in certain disease conditions, when a disruption in the tight junctions leads to an increased paracellular permeability (Mikitsh and Chacko 2014; Stamatovic et al. 2008). Another refinement of our model would consist of including cerebral blood flow, as this may be important for drugs that easily cross the BBB (Hammarlund-Udenaes et al. 2008; Banks 2009).

Extending the model to 3D is not only an important step in improving the realism of our model, but also a great possibility to study the drug distribution within the brain on a larger scale, by connecting multiple brain tissue units. This will increase the importance of the local concentration profiles of a drug within a brain tissue unit, as this brain tissue unit will be part of a larger framework in which the drug is spatially distributed. This will allow us to implement regional differences by assigning different parameter values to different brain tissue units, e.g. drug exchange with the blood capillaries could be different per capillary or binding sites could be unevenly distributed over space. This gives interesting possibilities, such as mimicking the effects of a local disease on the spatial concentration profiles of a drug. The 2D model we have now developed is an essential step in setting up a 3D mathematical model on the spatial distribution of drugs within the brain.

## Appendix I: Non-dimensionalisation of the Model

We have made the system of Eqs. (3–4) dimensionless by introducing a change in variables. Here, the original variables are scaled to dimensionless variables by scaling with a characteristic, dimensional scale. We set:

$$\begin{aligned} \textit{t}&= t_c \tau&\textit{D*}&= D_c d&k_{\mathrm {1on}}&= k_{\mathrm {1on_c}} K_{\mathrm {1on}} \\ \textit{x}&= x_c \xi ,&\textit{v}&= v_c V&k_{\mathrm {1off}}&= k_{\mathrm {1off_c}} K_{\mathrm {1off}} \\ \textit{y}&= y_c \eta&B_{\mathrm {1}}^{\mathrm {max}}&= B_{\mathrm {1_c}}^{\mathrm {max}} b_{\mathrm {1}}^{\mathrm {max}}&k_{\mathrm {2on}}&= k_{\mathrm {2on_c}} K_{\mathrm {2on}} \\ C_{\mathrm {ECF}}&= C_c u&B_{\mathrm {2}}^{\mathrm {max}}&= B_{\mathrm {2_c}}^{\mathrm {max}} b_{\mathrm {2}}^{\mathrm {max}}&k_{\mathrm {2off}}&= k_{\mathrm {2off_c}} K_{\mathrm {2off}} \\ B_1&= B_{1_c} b_1&C_{\mathrm {pl}}&= C_{\mathrm {pl_c}} w&P&= P_c p \\ B_2&= B_{2_c} b_2 \\ \end{aligned}$$

where

$$\begin{aligned} t_c&= 1s&D_c&= 10^{-10} \, \mathrm {m^2s^{-1}}&k_{\mathrm {1on_c}}&= (\upmu \mathrm {mol}\,\mathrm {L}^{-1}\,\mathrm {s})^{-1}\\ x_c&= 10^{-6}\, \mathrm {m}&v_c&= \mathrm {10^{-6} \, \mathrm {m\, s^{-1}}}&k_{\mathrm {1off_c}}&= \mathrm {10^{-2}\, s^{-1}}\\ y_c&= 10^{-6}\,\mathrm {m}&B_{\mathrm {1_c}}^{\mathrm {max}}&= \mathrm { \upmu mol \, L^{-1}}&k_{\mathrm {2on_c}}&= 10^{-2} \, (\upmu \mathrm {mol}\,\mathrm {L}^{-1}\,\mathrm {s})^{-1} \\ C_c&= \upmu \mathrm {mol \, L^{-1}}&B_{\mathrm {2_c}}^{\mathrm {max}}&= \mathrm {\upmu mol \, L^{-1}}&k_{\mathrm {2off_c}}&= \mathrm {s^{-1}} \\ B_{1_c}&= \upmu \mathrm {mol}\, \mathrm {L^{-1}}&C_{\mathrm {pl_c}}&= \mathrm {\upmu mol \, L^{-1}}&P_c&= 10^{-9}\, \mathrm {m\, s^{-1}} \\ B_{2_c}&= \mathrm {\upmu mol \, L^{-1}} \end{aligned}$$

This leads to the following system of dimensionless equations

$$\begin{aligned} \frac{{\partial } u}{{\partial } \tau }&= \mathrm {10^{2}} d \left( \frac{\partial ^2 u}{{\partial } \xi ^2} + \frac{{\partial }^2 u}{{\partial } \eta ^2}\right) - V \frac{{\partial } u}{{\partial } \xi } \\&\quad - K_{\mathrm {1on}}u(b_{\mathrm {1}}^{\mathrm {max}}-b_1) + 10^{-2}K_{\mathrm {1off}}b_1 \\&\quad - 10^{-2} K_{\mathrm {2on}}u(b_{\mathrm {2}}^{\mathrm {max}}-b_2) + K_{\mathrm {2off}}b_2, \\ \frac{{\partial } b_1}{{\partial } \tau }&= K_{\mathrm {1on}}u(b_{\mathrm {1}}^{\mathrm {max}}-b_1) - 10^{-2} K_{\mathrm {1off}}b_1, \\ \frac{{\partial } b_2}{{\partial }\tau }&= 10^{-2} K_{\mathrm {2on}}u(b_{\mathrm {2}}^{\mathrm {max}}-b_2) - K_{\mathrm {2off}}b_2. \end{aligned}$$

The corresponding boundary conditions are given by

$$\begin{aligned} d \frac{\partial u}{\partial \xi } = 10^{-5}{p} (w-u)(\xi , \eta ,\tau ) \end{aligned}$$

for $\xi =1$ and $\xi =0$ and

$$\begin{aligned} d \frac{\partial u}{\partial \eta } = 10^{-5}{p} (w-u)(\xi , \eta ,\tau ) \end{aligned}$$

for $$\eta =1$$ and $$\eta =0$$.

The initial conditions become

$$\begin{aligned} u (\xi , \eta , \tau =0)=0. \end{aligned}$$
